# Effects of ascorbic acid on intestinal flora and metabolites of C57 mice exposed to formaldehyde in digestive tract

**DOI:** 10.1371/journal.pone.0336977

**Published:** 2025-11-19

**Authors:** Xin Ling, Ziyan Hao, Yixuan Shi, Yuting Li, Kehan Wang, Yunshan Zhang, Yue Wang

**Affiliations:** 1 Department of Human Anatomy and Cytoneurobiology, School of Basic Medicine, Soochow University, Suzhou, P.R. China; 2 Experimental Teaching Center for Clinical Skills, Experimental Center, Medical College, Soochow University, Suzhou, China; Yantai Institute of Technology, CHINA

## Abstract

The diversity of microbiota and metabolites plays a key role in regulating metabolism, host immune response, neurobehavioral effects and detoxification mechanism in the digestive tract gut. Formaldehyde (FA) affects the gastrointestinal tract and its microbiota, whereas ascorbic acid (VC) improves gut health and selectively promotes microbial growth. In this study, we employed 16S rRNA sequencing and non-targeted metabolomics approaches to investigate these interactions. Our results demonstrated that Lachnospiraceae_NK4A136_group, Lactobacillus, Ligilactobacillus, Clostridiales_unclassified, and other microflora significantly decreased following FA exposure, whereas the intestinal flora changed in the exact opposite way following VC administration. And compared with FA group, the number of 492 ions were regulated, in which 382 feature was up-regulated and 304 feature was down-regulated in FA + 150 mg VC group. In addition, a correlation between gut microbiota and metabolites was observed. These results reveal the effects of FA or VC on the gastrointestinal tract and its microbiota, and our understanding in the treatment of FA-induced damage to the digestive tract.

## Introduction

The different groups of bacteria carried in the human gastrointestinal tract are collectively referred to as intestinal microbiota [1]. There are great differences between the bacteria observed in different individuals, but the main phyla remain unchanged within the species [2]. Intestinal microflora can affect host development and health by regulating metabolism, host immune response, neurobehavioral effects and detoxification mechanism, and play an important role in mental illness and other diseases [3]. For example, intestinal microflora communicates with the brain through cytokines released by mucosal immune cells and hormones produced by endocrine cells or through the vagus nerve. On the contrary, stress-related signals such as stress hormones or sympathetic neurotransmitters (GABA, 5-HT precursors, etc.) can affect intestinal microflora, physiology, gastrointestinal secretion and motility [4]. In recent years, some progress has been made in the composition and metabolism of human microflora, which has an important impact on human health [5]. However, the ecological imbalance of intestinal flora can lead to digestive system dysfunction and a variety of diseases, including obesity, inflammatory bowel disease, diabetes, allergies, arthritis and cardiovascular diseases [6].

Colorless and poisonous, FA poses a major threat to human health. Strong mucous membrane irritation and tear-inducing properties of FA can result in a variety of symptoms, including coughing, acute and chronic bronchitis, skin allergies, nasopharyngeal discomfort, conjunctival congestion and inflammation, nausea, vomiting, and gastrointestinal disorders. At present, it has been found that FA can induce changes in intestinal microflora in mice, which is characterized by changes in the abundance and diversity of intestinal microflora, and some KEGG pathways will also be affected by FA exposure [7].

Accumulating evidence suggests that vitamins can directly regulate the gut microbiota when given a vitamin that exceeds the upper limit of absorption in the small intestine which gives it a sufficient dose to reach outside the small intestine or in the form of a colon-targeted delivery system. Additionally, vitamins can impact gut microbiota through the systemic circulation indirect method, which has a direct bearing on human health [8]. Numerous metabolites may be involved in the various intestine redox regulation processes. VC can selectively stimulate microbial development by altering the redox potential to improve the circumstances in the cavity [9]. It exhibits resistance to degradation or conjugation by a broad spectrum of bacteria, fungi, and viruses [10]. Furthermore, individuals with inflammation bowel disease (IBD) had varying levels of reduced and oxidized VC, which may promote the differentiation of inflammatory T cell subtypes [11].

VC can partially counteract the harmful effect of digestive tract FA exposure on mice according to our findings, which may change the microbiota and metabolites in the digestive tract gut of mice. Therefore, metabolite analysis and 16s rDNA sequencing were used to examine the effects of FA and VC exposure.

## Materials and methods

### Experimental animal, model establishment and ethics statement

#### Experimental animal.

The C57 mice, provided by the Animal Center of Soochow University, used in the experiment were all females, weighing 18–20 g, and kept in an SPF-level animal room with a constant temperature and sterile environment at 25°C and a 12-hour diurnal cycle.

#### Model establishment.

All experimental mice were first acclimated to the environment for 1 week. The WT group (control) received normal saline by gavage. The FA group and FA + 150mgVC group were given 200 mg/kg formaldehyde (FA) by gavage (schedule: 3 days of gavage every 4 days, cycling twice) to establish the digestive tract FA exposure model. Additionally, the FA + 150mgVC group was intraperitoneally exposed with 150 mg/kg ascorbic acid (VC) immediately after each FA gavage.

#### Ethics statement.

1) Analgesia and Efforts to Alleviate Suffering: To reduce mouse discomfort, targeted welfare measures were applied. Trained personnel performed oral gavage (18-gauge rounded-tip needles, less esophageal irritation) and intraperitoneal injection (26-gauge needles, lower abdominal quadrants to avoid vital organs). Post-procedure, mice were observed individually for 30 minutes to check for adverse reactions (e.g., retching, injection-site bleeding); daily assessments included body weight (every 2 days), food/water intake and activity. Mice with >10% weight loss or persistent distress (e.g., hunched posture) were evaluated by a veterinarian, with early euthanasia as needed. Cages (3–4 same-group mice) had sterile nesting materials and chew sticks to reduce stress. Predefined humane endpoints (approved by Soochow University’s Experimental Animal Ethics Committee) for early termination included >15% initial weight loss, inability to access food/water, 24 + hours of pain behaviors, or severe tissue damage. 2) Mice were euthanized via CO₂ inhalation (medical-grade) to minimize distress. CO₂ was delivered into a sealed, ventilated chamber (calibrated flowmeter, 50–70% volume displacement/min) to avoid panic. After breathing ceased (3–5 minutes), mice remained for 5 more minutes to confirm death—verified by no heartbeat (gentle chest palpation) and no corneal reflex (no blinking to light swab touch). All steps complied with the committee’s regulations. 3) All animal experiments in this study were approved by the Experimental Animal Ethics Committee of Soochow University, and all operations involved in animal experiments were in accordance with the regulations of the Experimental Animal Ethics Committee of Soochow University.

### HE staining

Mouse intestinal tissue was taken to make paraffin sections, and then deparaffinized, hematoxylin staining, eosin staining, and mounting were made into HE stained longitudinal section specimens.

### 16S rDNA

The total DNA of microbiome samples from various sources was extracted using the CTAB method. Agarose gel electrophoresis was used to assess the quality of the DNA extraction, and an ultraviolet spectrophotometer was used to quantify the extracted DNA. The genome DNA template was used for PCR with primers in S1 Table. PCR products were purified by AMPure XT beads and quantified by Qubit. PCR amplification products were detected by agarose gel electrophoresis, and the gel was recovered by AMPure XT beads recovery kit. The qualified PCR products were mixed according to the required amount of sequencing, and then denatured with NaOH and sequenced on the computer. The raw data sequences were processed into clean data sequences. Alpha diversity was evaluated using the Chao1 and Shannon indices, while beta diversity was analyzed via principal coordinate analysis (PCoA) based on Bray-Curtis distances. All analyses were performed using QIIME2 software to ensure methodological clarity. The changes of abundance in intestinal microbiota among each group were observed with species analysis. The taxonomic assignments were primarily resolved to the genus level. The differential changes in intestinal microbiota among each group were observed with analysis of the difference.

### Non-targeted metabolomics

After the mouse model was constructed, the mice were euthanized using CO_2_. Within 10 min, they were moved to a UV-sterilized biosafety cabinet. The abdomen was disinfected; the small intestine was dissected, rinsed with pre-cooled sterile NaCl, and placed on ice. Contents were squeezed into a pre-cooled enzyme-free tube. The metabolites were extracted with 1 ml 50% methanol (precooled) per 100 mg sample, and then incubated at room temperature for 10 minutes after vortex 1 min; the extracted mixture was stored at-20 °C. All the samples are collected by the LC-MS system according to the machine instructions. All chromatographic separation uses Vanquish Flex ultra-high performance liquid chromatography system. ACQUITYUPLCT3 column is used for reversed phase separation. High resolution tandem mass spectrometer (Q-Exactive) was used to detect the metabolites eluted from the chromatographic column. Q-Exactive operates in both positive and negative ion modes. The precursor spectrum (70-1050m/z) is collected at a resolution of 70000 to achieve the goal of AGC3e6. The maximum injection time is set to 100ms. In DDA mode, the first three configurations are used to get the data set to DDA mode. The fragment spectrum was collected at a resolution of 17500 to reach the target value of AGC. The raw data was converted into mzXML formation with MSConvert software. And xcms software were used for Peak extraction. The metabolites were annotated based on database of KEGG and HMDB. The resulting data was imported into mateX. Then we removed missing values, filled in the missing values with KNN algorithm, did data normalization and removal of unstable metabolites in order to make metabolite quantification. T test was used to do univariate analysis. Principle component analysis (PCA) and Partial least squares discrimination analysis (PLS-DA) were used to do multivariate analysis. The Significantly differential metabolites were selected based on ratio>2, Variable importance of projection (VIP) values (VIP > 1.0), q < 0.05.

### Joint analysis

This study explored the interaction between microorganisms and their metabolites by metabonomic analysis and 16s sequencing analysis. Through the Spearman correlation analysis of the differential secondary metabolites obtained by metabolomics and the significant differences obtained by 16s sequencing analysis, the relationships between differential bacteria and differential microflora, differential metabolites and differential metabolites, differential metabolites and differential microflora were obtained.

## Results

### Diversity analysis of intestinal microflora

**Fig 1:**
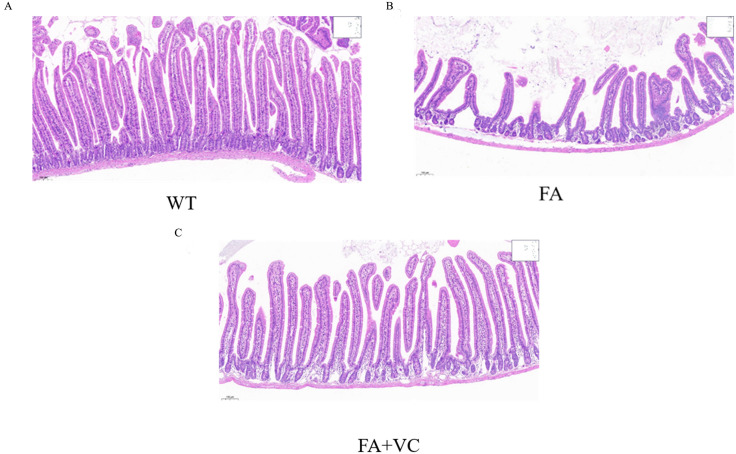
Effect of VC on the pathologic structure of the small intestine in the digestive tract exposed to FA of mice. **(A)** HE staining of longitudinal sections of mouse small intestine in WT group. **(B)** HE staining of longitudinal sections of mouse small intestine in FA group. **(C)** HE staining of longitudinal sections of mouse small intestine in FA + 150 mg VC group.

The structural abnormalities in the small intestine and altered behavioral ability of mice were induced by FA exposure in the digestive tract. To conform whether VC could antagonize the pathological changes induced by FA exposure, the intestinal tissues was observed through HE staining. The results showed that compared with WT group (Fig 1A), the number of intestinal villi was reduced and the gaps of villus were widened in FA group (Fig 1B), which means that the barrier of small intestine was impaired in the FA group. And the abnormal changes were improved in FA + 150 mg VC group (Fig 1C).

**Fig 2:**
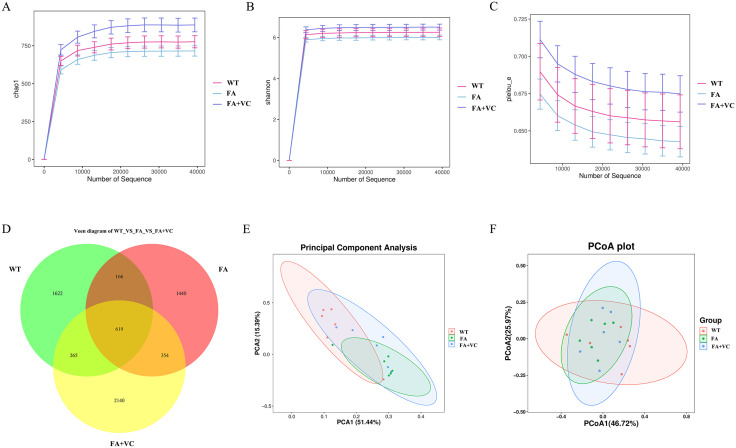
Analysis of alpha and analysis of beta diversity of intestinal flora. **(A)** Chao and observed_species are the number of species contained in the estimated sample. **(B)** The higher the Shannon index, the higher the species diversity. **(C)** Pieloue reflects the evenness, and the larger the value, the more uniform the species. **(D)** Venn diagram, reflecting the common and unique number of ASV for all samples/ groups. **(E)** PCA diagram, the more similar the species composition of the sample, the closer their distance in the PCA diagram. **(F)** PCoA diagram, the closer the sample distance is, the smaller the difference is, and the more similar the structure is.

Then intestinal contents of mice in different groups were used for 16srDNA and non-targeted metabolism detection. Alpha diversity analysis showed that the sparse curve chao1 of all different samples reaches a platform, indicating that the number of samples used in sequencing meets the requirements. The Goods_coverage value was 1, which indicated that the sequencing results were true and reliable. As Fig 2A-C, the number, abundance and evenness of intestinal microflora were lower in FA group than those in WT, while that in FA + 150mgVC group was higher than that in FA group. These results suggested that the number, abundance and evenness of intestinal microflora were decreased in FA exposed, while that significantly increased after administration of VC.

The results of Venn diagram (Fig 2D) showed that 2672 ASV numbers were obtained in WT group, 2579 ASV numbers in group FA and 785 ASV numbers in both groups, indicating that FA significantly changed the species of intestinal microbiota in mice. While 3378 ASV numbers were obtained in FA + 150mgVC group, which 973 ASV numbers were found in FA group, indicating that VC further changed and expanded the number of species of intestinal microflora. Based on the sample species abundance table (Euclidean distance) dimension reduction order, the intra-group similarity and inter-group difference of biological repetition were obvious, through PCA analysis (Fig 2E), which were consistent with The PoCA diagram (Fig 2F).

### Abundance of bacterial families of intestinal microflora

**Fig 3:**
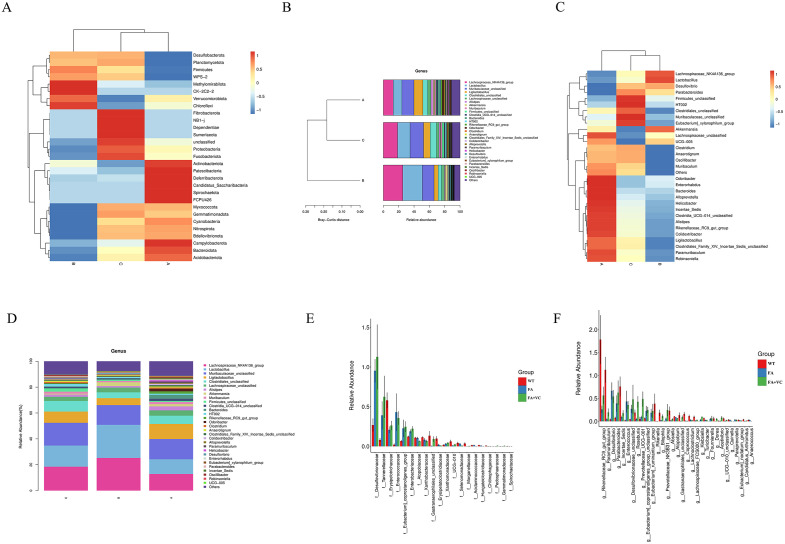
Analysis of Species and analysis of the difference of intestinal flora. **(A)** Each row of the phylum heat map represents the species and each column represents the sample/group. **(B)** Genus Bray-Curtis distance clustering tree structure. **(C)** Genus heat map. **(D)** Genus stacked bar chart. **(E)** The significant difference of intestinal microflora in family level was analyzed, and the differences of all species at each level were analyzed. **(F)** Analysis of the significant difference of intestinal microflora at Genus level.

According to the relative abundance table of species, the communities with the top 30 relative abundance of species with different classification levels were selected to cluster, and the results were presented by heat map (Fig 3A). We observed that compared with WT group, the abundance of Firmicutes, Verrucomicrobiota, Desulfobacterota and Campylobacterota population were increased, while the abundance of Bacteroidota, Proteobacteria and Actinobacteriota were decreased in FA group at the Phylum level. Compared with FA group, the abundance of Firmicutes, Verrucomicrobiota, Actinobacteriota, and Campylobacterota were decreased and the population of Bacteroidota and Proteobacteria were increased in FA + 150 mg VC. The relative abundance of Firmicutes and Verrucomicrobiota in FA group were higher, while Bacteroidota in FA + 150 mg VC group was relatively high. Among them, the change of Firmicutes and Bacteroidota were consistent with the reported changes of intestinal flora in DSS colitis mouse model.

Then the samples were clustered by Bray-Curtis distance at genus level. The results of cluster analysis showed that the species similarity between WT group and FA + 150 mg VC group was relatively high (Fig 3B). The results of heat map showed that there were significant differences in intestinal flora among WT, FA and FA + 150 mg VC groups (Fig 3C). The results of genus horizontal column stacking chart showed that compared with WT group, the abundance of Lachnospiraceae_NK4A136_group, Lactobacilus, Akkermansia were increased and the number of Ligilactobacillus, clostridiales_unclassified and Rikenellaceae_RC9_gut group were decreased significantly in FA group (Fig 3D). Compared with FA group, the abundance of Muribaculaceae_unclassified, Ligilactobacillus, Clostridiales_unclassified and Muribaculum, were increased, while Lachnospiraceae_NK4A136_group, Lactobacillus, Lachnospiraceae_unclassified and Akkermansia were decreased in FA + 150 mg VC group.

### Analysis of the difference of intestinal flora

In the classification of Family (Fig 3E), 19 intestinal flora were significant different between WT and FA group, in which f__Gastranaerophilales_unclassified，f__Erysipelotrichaceae，f__Morganellaceae， and so on were downregulated, while f__Desulfovibrionaceae，f__Enterococcaceae，f__Hafniaceae and so on were upregulated. 10 intestinal flora were significantly different between FA and FA + 150 mg VC group. The relative abundance of intestinal microflora such as f__Bifidobacteriaceae， f__Selenomonadaceae，and so on was downregulated, while f__Pedosphaeraceae， f__Brucellaceae，f__Gastranaerophilales_unclassified and f__Gemmatimonadaceae was upregulated.

In genus classification (Fig 3F), 37 intestinal flora were significantly different between WT group and FA group, and 20 intestinal flora were significantly different between FA + 150 mg VC group and FA group.

### Advanced analysis and functional enrichment profiles of intestinal differential flora

**Fig 4:**
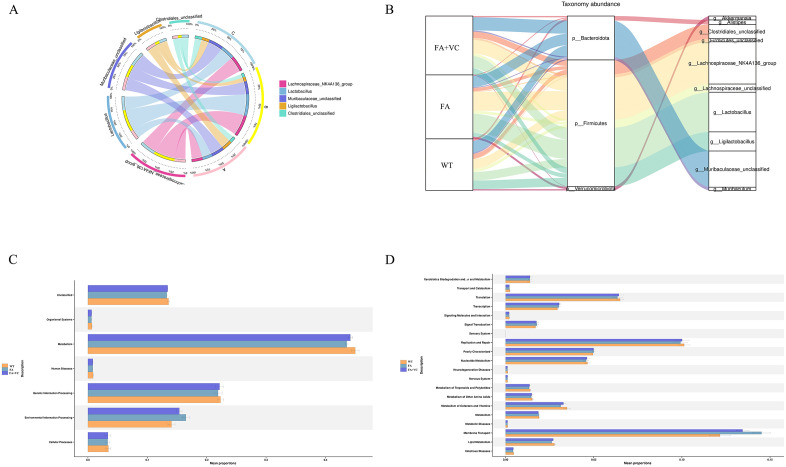
Advanced analysis and functional enrichment profiles of the difference of intestinal flora. **(A)** Genus level Advanced Analysis. **(B)** Sanji diagram and the species has a higher abundance in the red group. **(C/D)** Function prediction chart.

In the classification of intestinal microflora (Phylum) and genus (Genus), we found that there were significant differences among the three groups. The dominant flora has different definitions in different projects. In genus classification (Fig 4A), we found that the abundance of Lachnospiraceae_NK4A136_group, Lactobacillus in FA group was significantly higher than that in WT group, while the abundance of Ligilactobacillus and Clostridiales_unclassified in FA group was significantly lower than that in WT group. The abundance of Lachnospiraceae_NK4A136_group, Lactobacillus in FA + 150 mg VC group was significantly lower than that in FA group, while Ligilactobacillus and Clostridiales_unclassified, Muribaculaceae_unclassified were significantly higher in FA + 150 mg VC group.

Through the Sangji diagram (Fig 4B), we found that the intestinal flora of each group were mainly concentrated in three phyla of P_Firmicutes, P_ Bacteroidota and P_Verrucomicrobiota, and 10 genera, such as g_Akkermansa, g_Alistipes, g_Clostridiales_unclassified, g_Firmicutes_unclassified, g_Lachnospiracese_unclassified, g_Lachnospiracese_NK4A136_group, g_Lactobacillus, g_ Ligilactobacillus, g_Muribaculace_unclassified，g_ Muribaculum and so on.

Based on the predicted results of PICRUSt2 function (Fig 4C-D), we speculate that the differences in intestinal microbiota among different groups may be related to three main pathways such as metabolism, genetic information processing, environmental information processing, which may be related to cell membrane transport, transcriptional translation, replication repair, lipid metabolism and so on.

### Diversity and difference analysis of metabolites

**Fig 5:**
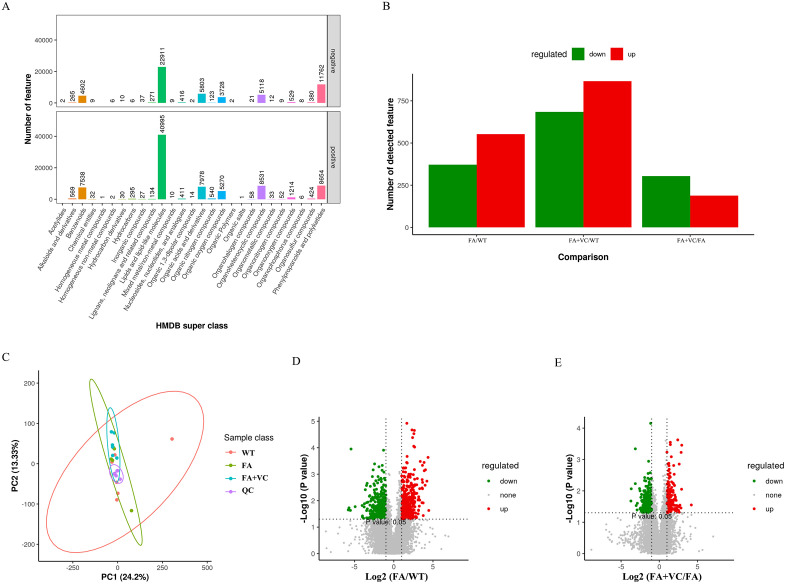
Diversity and difference analysis of metabolites. **(A)** HMDB Super class classification chart. **(B)** Statistical histogram of differential metabolic ions. **(C)** PCA analysis. **(D)** (FA vs WT) Volcanic map. **(E)** (FA + VC vs FA) Volcanic map.

In order to further clarify the changes of intestinal microflora metabolites, the composition and biological function of metabolites were explored by non-targeted metabolomics. The results of HMDB Super class classification chart showed that Lipids and lipid-like molecules, Benzenoids, Organic acids and derivatives, Phenylpropanoids and polyketides, Organoheterocyclic compounds had the highest number of negative and positive metabolites (Fig 5A).

We found that there were significant differences in metabolic ions among the groups by PCA analysis (Fig 5C). In the further study, the differentially expressed metabolites in different groups were screened by univariate analysis and T-test (Fig 5B). The results showed that compared with WT group, the number of positive feature in FA group was down-regulated and up-regulated by more than 200 positive feature, and the number of negative feature was down-regulated by more than 100 negative feature and increased by less than 300 negative feature compared with WT group. The results also showed that compared with FA group, the number of positive feature in FA + 150 mg VC group was down-regulated and up-regulated by more than 100 positive feature, and the number of negative feature was down-regulated by more than 100 negative feature and increased by less than 100 negative feature compared with FA group.

The differential metabolites were showed by the volcanic map (Fig 5D-E). With WT group, the number of 925 ions were regulated, in which 553 feature was up-regulated and 372 feature was down-regulated in FA group. While compared with FA group, the number of 492 ions were regulated, in which 382 feature was up-regulated and 304 feature was down-regulated in FA + 150 mg VC group.

### Functional analysis of metabolites

**Fig 6:**
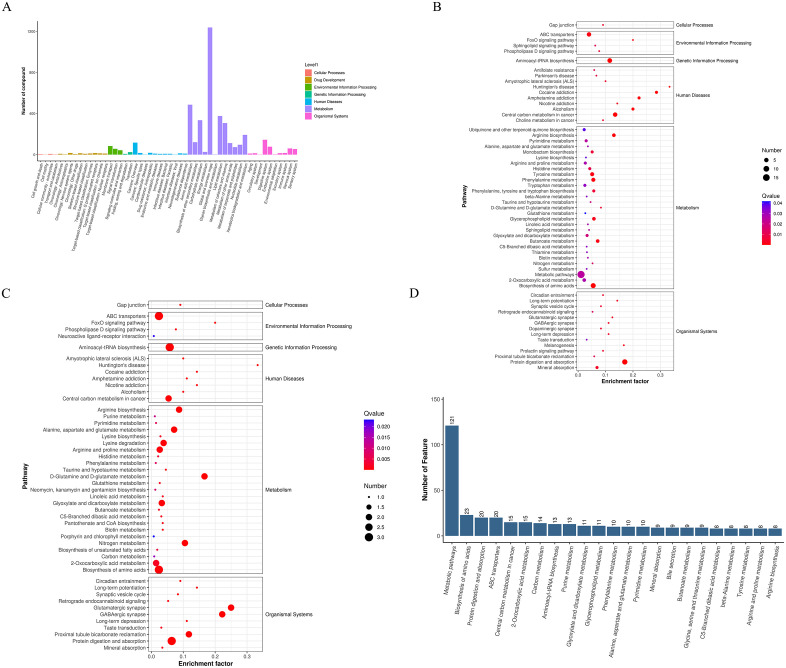
Functional analysis of metabolites. **(A)** KEGG pathway classification chart. **(B)** (FA vs WT) differential metabolites KEGG bubble diagram. **(C)** (FA + VC vs FA) differential metabolite KEGG bubble diagram. **(D)** Metabolic pathway.

The KEGG pathway classification diagram of primary metabolites (Fig 6A) shows that the metabolites in the samples are mainly related to Amino acid metabolism; Carbohydrate metabolism; Global and overview maps; Lipid metabolism; Metabolism of cofactors and vitamins and other signal pathways. (Fig 6B-C) The pathways of secondary metabolites are mainly concentrated in Drug development, environmental information processing, genetic information processing, human diseases, metabolism, organism system and mainly related to ABC transporters, aminoacyl-tRNA biosynthesis, metabolic pathways, Central carbon metabolism in cancer, biosynthesis of amino acids, protein digestion and absorption and so on.

In order to further explore the function of metabolites, the Top 20 Pathway entry is Abscissa and the corresponding item metabolite quantity is ordinate. We found that metabolites (Fig 6D) are mainly related to Metabolic pathways, Biosynthesis of amino acids, Protein digestion and absorption, ABC transporters, Central carbon metabolism in cancer and other pathways.

### Association between intestinal microbes and metabolites

**Fig 7:**
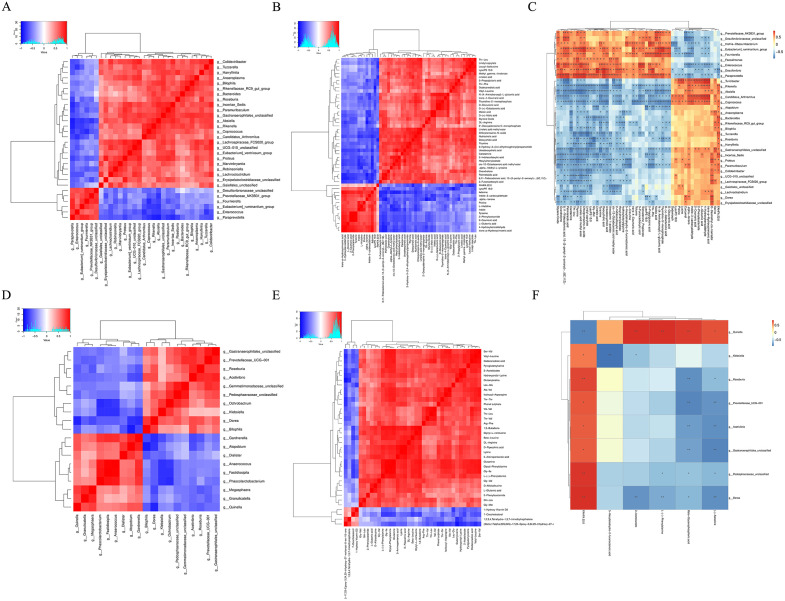
Correlation between differential flora and differential metabolites. **(A)** (FA vs WT) Correlation between differential flora and differential flora. **(B)** (FA vs WT) Correlation between differential metabolites and differential metabolites. **(C)** (FA vs WT) Correlation between differential microflora and differential metabolites. **(D)** (FA + VC vs FA) Correlation between differential flora and differential flora. **(E)** (FA + VC vs FA) Correlation between differential metabolites and differential metabolites. **(F)** (FA + VC vs FA) correlation between differential microflora and differential metabolites (^*^p < 0.05; ^**^ p < 0.01).

The interaction between microorganisms and their metabolites was explored. The results showed that there was significant positive and negative correlation between differential flora and differential flora, and there was also a significant correlation between differential metabolites and differential flora between the two groups (Fig 7A-C). Between FA group and FA + 150 mg VC group (Fig 7D-F), we also observed significant correlations between different microflora and differential metabolites, differential metabolites and differential flora.

## Discussion

Intestinal microbiota is linked to many human disorders, including inflammatory bowel disease (IBD) [12], irritable bowel syndrome (IBS) [13], diabetes [14], allergy diseases, and others. Environmental factors may influence the composition of intestinal microflora. There is a close association between human diseases, intestinal microbiota, and exposure to pollutants like FA.

Here, we collected the intestinal contents of mice across various treatment groups in order to detect 16srDNA and non-targeted metabolism. We discovered that FA decreased the population size, diversity, and evenness of the mouse intestinal microbiota. Following VC treatment, there was a notable improvement in the decreased microflora’s quantity, evenness, and abundance. Furthermore, FA’s impact on mice’s intestinal flora was comparable to that of other environmental contaminants such heavy metals or polychlorinated biphenyls (PCBs). Modifications in the intestinal microbiota of mice may worsen the harm caused by FA.

The species composition of various samples (groups) was examined in accordance with the species abundance table of various levels, and Bacteroides dominated the intestinal microbiota of each group, which was in line with the findings of other studies [15]. Nine bacteria had modifications at the gate Phylum level, and the effects of VC delivery on gut flora were found to be opposite those of FA exposure. Supplementing with VC effectively mitigated this increase in F/B to Bacteroides B ratio, which was in line with the findings of animal models fed a high-fat diet.

Studies have revealed that Campylobacter, one of the bacteria with a higher relative abundance following FA treatment, has genotoxic, anti-inflammatory, and anti-tumor effects on the host. Desulfurization bacteria, on the other hand, can trigger an intestinal immune response and result in experimental colitis [16]. The two above bacteria were increased after FA’s damage. Bacteroides, may impact host neurodevelopment by modifying intestinal barrier integrity and the availability of metabolites, including synthetic short-chain fatty acids (SCFA), like butyrate, propionate, and acetate [17]. Here, Bacteroides was decreased relative abundance following FA exposed.

These findings demonstrate that gut microbiota is negatively impacted by FA and that this effect is dose-dependent. VC effectively restored the harm caused by FA by altering the kind and quantity of intestinal flora, which is involved in metabolism, neurodevelopment, and other aspects of health.

Furthermore, we concentrate on the top five plants in abundance out of a total of fifteen genera. Lachnospiraceae_NK4A136_group, Lactobacillus, Ligilactobacillus, Clostridiales_unclassified, and other microflora significantly decreased following FA exposure, whereas the intestinal flora changed in the exact opposite way following VC administration. Lachnospiraceae_NK4A136_group is one of the bacteria that FA causes to have a greater relative abundance. One potential role for lactobacillus is the restoration of the gut mucosal barrier [18]. In the large intestine, clostridiales can break down dietary fiber to create short-chain fatty acids and preserve intestinal health [19]. The gut flora muribaculaceae, which is linked to tryptophan metabolism and has the ability to create butyrate, is more abundant in the population when exposed to VC [20]. The aforementioned findings demonstrate that exposure to FA alters the quantity and variety of intestinal microflora, the majority of which are connected to inflammation and SCFAS synthesis. VC regulation of the gut flora was nearly the opposite of FA regulation, which significantly lessened the harm from FA exposure.

We investigated the metabolite composition and biological functions in the samples using non-targeted metabolomics and high-resolution mass spectrometry to better understand the changes in gut microbiota metabolites in each group. The KEGG pathway results for the first-level metabolite showed that the metabolites were primarily associated with the metabolism of amino acids, carbohydrates, lipids, cofactors, and vitamins. The HMDB Super class classification chart revealed that the number of lipid and lipid molecular metabolites was the largest, up to 63906. It mostly affects cancer metabolism, central carbon metabolism, protein digestion and absorption, amino acid biosynthesis, and ABC transporter. These findings support the gut microbiota hypothesis, indicating that FA and VC may control different physiological processes in the host by means of gut microbiota metabolites.

For Spearman correlation analysis, the substantial differences found by 16-sequence sequencing analysis and the differential secondary metabolites identified by metabolomics screening belong to the horizontal flora. The findings demonstrated a strong positive and negative correlation between differential metabolites and differential microflora, as well as a significant correlation between differential metabolites and differential microflora between the FA group and the Control group. Likewise, notable distinctions were observed between the FA + 150mgVC group and the FA group.

This study primarily focuses on the crosstalk between VC, FA, and gut microbiota-metabolites, so it does not include analyses of intestinal epithelial function and multi-segment histology, nor fecal/cecal omics data—these are limitations to be addressed. Future research will systematically evaluate epithelial cell differentiation using markers like villin and lysozyme, detect mucus production (e.g., MUC2), and perform HE staining on all intestinal segments to clarify tissue-level perturbations. Additionally, integrating fecal/cecal proteomics and transcriptomics will help delineate regulatory networks governing metabolite changes, thereby filling the knowledge gaps highlighted in this review and providing more comprehensive insights into gut homeostasis modulation.

In summary, VC could improve the intestinal injury after FA exposed by altering the content of its metabolites and intestinal flora to the host’s physiological function to alleviate gut damage.

## Supporting information

S1 Table The sequences of primers. The left column showed the gene names. The right column showed the corresponding sense and antisense sequences of Primers.
(TIF)

## Abbreviations

FA: Formaldehyde
VC: ascorbic acid
IBD: inflammation bowel disease
IBS: irritable bowel syndrome
PCBs: polychlorinated biphenyls
SCFA: short-chain fatty acids
GO: Gene Ontology
KEGG: Kyoto Encyclopedia of Genes and Genomes
HIBEC: human intrahepatic biliary epithelial cells
PCR: Polymerase Chain Reaction
HE: hematoxylin-eosin
