# Supplementary figures and images for: Effects of ascorbic acid on intestinal flora and metabolites of C57 mice exposed to formaldehyde in digestive tract
Source: PLoS One. 2025 Nov 19;20(11):e0336977. doi: 10.1371/journal.pone.0336977 (PMC12629445; doi:10.1371/journal.pone.0336977)

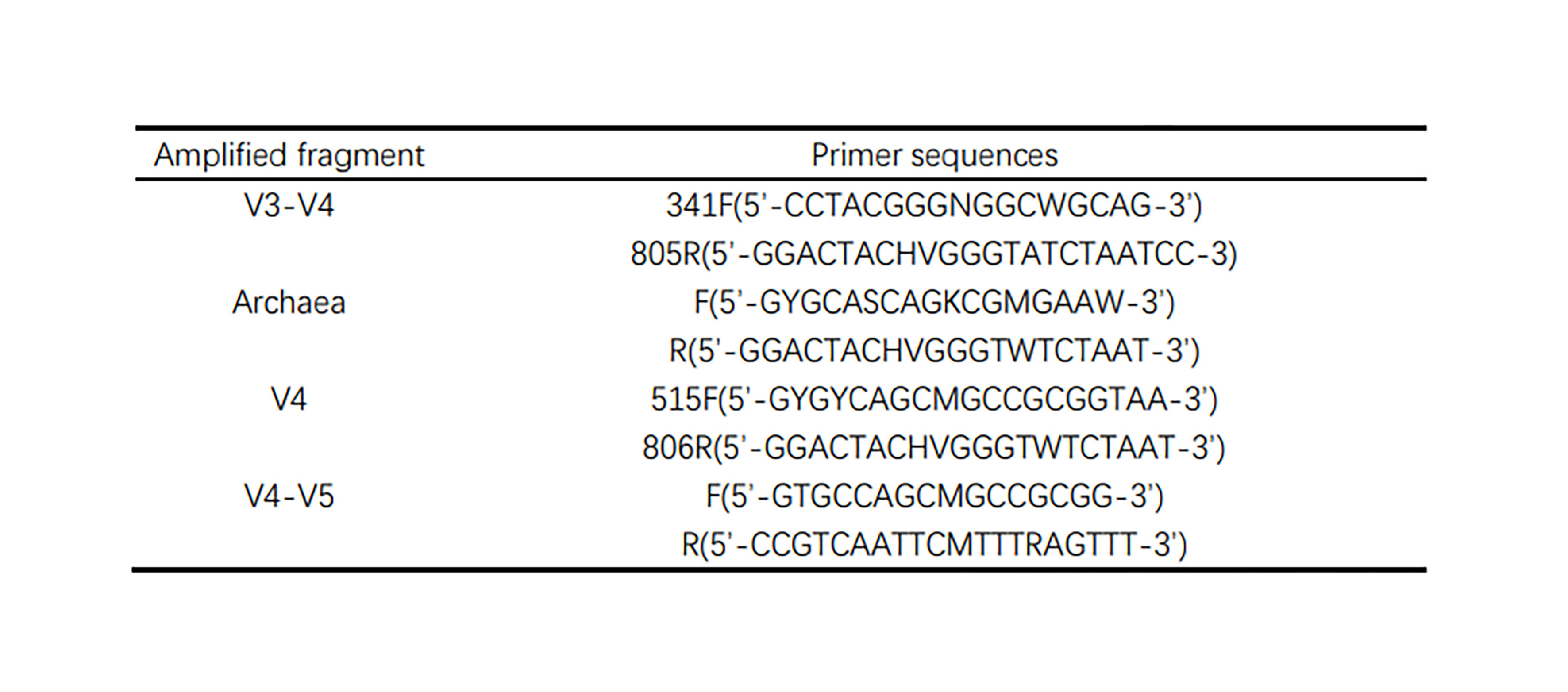

Supplement: S1 Table — The left column showed the gene names. The right column showed the corresponding sense and antisense sequences of Primers. (TIF) [file pone.0336977.s001.tif]
